# P2 Receptors as Therapeutic Targets in Allergic Rhinitis: Insights From the Use of Natural Products and Preclinical Studies

**DOI:** 10.1155/jimr/7715676

**Published:** 2026-06-25

**Authors:** Thalita Calvet Pereira, Leandro Rocha, Robson Xavier Faria

**Affiliations:** ^1^ Laboratory of Environmental Health Assessment and Promotion, Oswaldo Cruz Institute (IOC), Oswaldo Cruz Foundation (Fiocruz), Rio de Janeiro, Rio de Janeiro, Brazil, fiocruz.br; ^2^ Postgraduate Program in Plant Biotechnology and Bioprocesses, Center for Health Sciences, Federal University of Rio de Janeiro (UFRJ), Rio de Janeiro, Rio de Janeiro, Brazil, ufrj.br; ^3^ Laboratory of Natural Products Technology, Fluminense Federal University (UFF), Niterói, Rio de Janeiro, Brazil, uff.br

**Keywords:** allergic rhinitis, natural products, NLRP3 inflammasome, P2 receptors, therapeutic targets

## Abstract

Allergic rhinitis (AR) is a chronic inflammatory disorder of the upper airways that is mediated by immunoglobulin E (IgE) and triggered by environmental allergens. Current pharmacological therapies, including antihistamines, corticosteroids, and immunotherapy, offer symptomatic relief but are limited by their incomplete disease‐modulating effects and potential adverse side effects. Purinergic P2 receptors (P2Rs; P2X and P2Y subtypes) have emerged as key modulators of allergic inflammation and regulate mast cell degranulation, T helper 2 (Th2) cytokine release, eosinophil recruitment, and NLRP3 inflammasome activation. Natural products, including polyphenols, flavonoids, terpenoids, and alkaloids, demonstrate multitarget anti‐inflammatory effects and have the potential to modulate P2R signaling. However, direct evidence of their activity on P2Rs in AR remains limited. This review critically summarizes the immunopathology of AR, highlights the functional relevance of P2Rs, and discusses emerging natural product‐based strategies, emphasizing mechanistic insights and translational potential for the development of novel therapeutics.

## 1. Introduction

Allergic rhinitis (AR) is a chronic inflammatory disease of the upper airways that is mediated by immunoglobulin E (IgE) and triggered by exposure to environmental allergens such as pollen, mites, and pollutants [1, 2]. It is characterized by symptoms such as nasal congestion, rhinorrhea, sneezing, itching, and nasal obstruction, which negatively affect patients’ quality of life [3]. With a growing prevalence in various parts of the world, including Brazil, this condition represents a significant public health issue because of both individual suffering and the associated socioeconomic impact [4]. Despite the availability of pharmacological therapies to control symptoms, such as antihistamines, intranasal and oral corticosteroids, and immunotherapies, many of these approaches have limitations because of undesirable side effects [5]. In this context, purinergic P2 receptors (P2R), including the P2X and P2Y subtypes, have emerged as important modulators of immune and inflammatory responses in the airways, influencing processes such as leukocyte recruitment, epithelial signaling, and cytokine release [6, 7]. Moreover, bioactive compounds derived from natural products have emerged as promising sources of new molecules with potential anti‐inflammatory and antiallergic effects [8].

This article aims to critically review the scientific evidence linking AR to P2R activation while also highlighting natural products with inhibitory effects on this receptor and highlighting innovative and translational therapeutic perspectives. A literature search was conducted in PubMed, Scopus, and Web of Science via the following Boolean operators: (“allergic rhinitis” OR “AR”) AND (“P2 receptor” OR “purinergic receptor”) AND (“natural products” OR “plant‐derived compounds”) AND (“anti‐inflammatory” OR “immunomodulatory”). Articles in English or Portuguese were included. Titles and abstracts were screened for relevance, and full texts were reviewed to extract data on P2R involvement in AR and the modulatory effects of natural products.

## 2. Pathophysiology and Immunology of AR

AR is defined primarily as a chronic inflammatory disease affecting the upper airways following exposure to a protein called an antigen or allergen [9]. The mechanism of AR results from a type I hypersensitivity reaction; an exaggerated immune response is triggered when the immune system of genetically predisposed or previously sensitized individuals recognizes antigens such as pollen, mites, or food, leading to the overproduction of specific IgE. To sensitize individuals, IgE binds to high‐affinity receptors (FcεRIs) on the surface of mast cells and basophils, priming these cells for subsequent exposure [10, 11].

**Figure 1:**
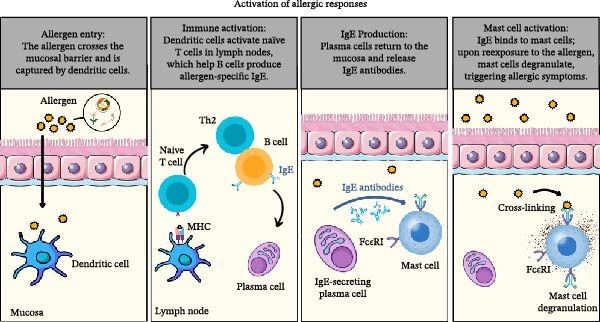
Schematic representation of allergic response activation. Allergens penetrate the mucosal barrier and are captured by dendritic cells, which migrate to lymph nodes and activate naïve T cells. T helper 2 (Th2) cells, which stimulate B cells to differentiate into plasma cells and produce allergen‐specific IgE, are activated. IgE antibodies return to the mucosa, where they bind to FcεRI receptors on mast cells. Upon reexposure to the allergen, cross‐linking of IgE–FcεRI complexes induces mast cell degranulation, releasing mediators that trigger allergic symptoms. Figure created with Canva licensed under CC BY 3.0 and adapted from Abbas et al. [11].

After the sensitization phase, upon reexposure to the allergen, cross‐linking occurs between antigen–IgE–mast cell complexes, leading to mast cell degranulation and the release of mediators such as histamine, prostaglandins, leukotrienes, and cytokines (Figure 1). This immediate phase is followed by a late inflammatory response characterized by the recruitment of eosinophils, T helper 2 (Th2) lymphocytes, and other inflammatory cells, which release cytokines such as IL‐4, IL‐5, and IL‐13 [12, 13]. The result is the maintenance of a persistent inflammatory environment, which is responsible for chronic symptoms and recurrent exacerbations and is frequently associated with other conditions, such as asthma and atopic dermatitis. This environment is also influenced by environmental factors and genetic susceptibility [14].

AR affects ~25%–40% of the population worldwide, depending on age, environment, and geographic region and is more prevalent in industrialized countries, where factors such as air pollution and lifestyle changes may contribute to its increase [15]. In Brazil, population and epidemiological studies indicate that ~30% of individuals report symptoms consistent with those of AR, representing a significant public health problem [16]. Although it does not pose a direct risk to life, its impact on sleep quality, work productivity, and academic performance is significant, and it is associated with an increased risk of developing asthma and chronic sinusitis [14].

### 2.1. Conventional Treatments and Their Limitations

The management of AR is primarily based on controlling symptoms and preventing immune responses using strategies that include allergen avoidance, pharmacotherapy, and, in some cases, specific immunotherapy. Conventional pharmacological treatments for allergies involve various approaches aimed at minimizing the symptoms triggered by the immune response, thereby reducing inflammation and preventing severe cases [17–19]. First‐line medication options include intranasal corticosteroids and H1 antihistamines (oral or intranasal), which may be used alone or in combination depending on the disease severity and symptom profile. H1 antihistamines are available in formulations for oral, intranasal, and ophthalmic use, and their main mechanism of action involves the antagonism of the histamine H1 receptor. Oral formulations are divided into first‐ and second‐generation drugs, which differ in their ability to cross the blood‒brain barrier (BBB), efficacy, and safety [20]. First‐generation agents, such as chlorpheniramine, can penetrate the CNS and control allergic symptoms but cause side effects such as sedation and the need for multiple daily doses and are not recommended for the treatment of AR [21]. Second‐generation agents, such as loratadine and cetirizine, have lower BBB penetration and prolonged half‐lives and are well tolerated, reducing central effects, demonstrating efficacy, and improving treatment adherence [22, 23]. Topically administered H1 antihistamines, such as olopatadine and azelestine, are generally considered second generation because they cause less sedation and have faster effects beginning at 1–3 h after administration, particularly because of their rapid onset of action and effectiveness in controlling nasal congestion, rhinorrhea, and sneezing. These agents may be used as monotherapies in mild cases or in combination with intranasal corticosteroids in moderate to severe diseases, resulting in enhanced symptom control [24, 25].

Additionally, oral and intranasal corticosteroids, as well as antileukotrienes, act on different molecular targets and are indicated according to the severity of the AR. According to major international guidelines, including the AR and its Impact on Asthma (ARIA 2020), intranasal corticosteroids are the most effective therapy for controlling overall inflammation; however, the same clinical guidelines recommend limiting their use because of the risk of adverse systemic effects, including metabolic and immunosuppressive complications. Therefore, their use should be restricted to short courses in carefully selected patients [14]. As glucocorticoids, they are widely used to manage severe allergic and inflammatory responses by inhibiting phospholipase A_2_, reducing proinflammatory gene expression, and suppressing prostaglandin synthesis via the inhibition of COX‐2 activity, thereby reducing the levels of inflammatory mediators and nasal mucosa edema. Intranasal forms, such as budesonide, act directly on the airways, providing an effective and safe anti‐inflammatory effect for prolonged use; however, caution is advised because of dose‐related and time‐related adverse effects. Antileukotrienes, such as montelukast, demonstrate moderate efficacy and are indicated as adjuncts in persistent asthma patients. Nasal or oral decongestants should be used for short periods because of the risk of rebound effects and cardiovascular events when they are used long‐term [26, 27]. Although available pharmacotherapies are highly effective at controlling AR, their continuous use is limited by side effects and the need for chronic administration, which can reduce treatment adherence. Furthermore, these drugs primarily provide symptomatic relief without altering the disease progression. Allergen‐specific immunotherapy, administered subcutaneously or sublingually, is the only intervention capable of modifying the disease course through long‐term immune modulation; however, it is limited by cost, prolonged treatment duration, and risk of adverse systemic reactions [2, 28].

These barriers justify the search for therapeutic alternatives that combine efficacy, safety, and better adherence. In this context, investigating new molecular targets and substances derived from natural products has emerged as a promising strategy for developing innovative approaches. A deep understanding of AR immunology has allowed the identification of new therapeutic targets, among which the purinergic pathway stands out, with P2Rs playing a key role in amplifying inflammatory responses (Figure 1) [29].

### 2.2. New Therapeutic Alternatives and Molecular Targets

Given the limitations of conventional treatments for AR, interest in developing more specific, safe, and effective therapies, including approaches based on natural products, is increasing. Recent studies have identified several molecular targets involved in modulating allergic immune responses, such as the NLRP3 inflammasome, the transcription factor NF‐κB, and proinflammatory cytokines, such as IL‐33, IL‐6, and IL‐9 [30, 31]. Several key mediators have been explored. For example, IL‐33 has been identified as a strong target owing to its presence in cells actively involved in the AR. IL‐33 binds to its membrane receptor ST2, also known as IL1RL1, triggering allergic signaling cascades; blocking the IL‐33–ST2 interaction is a strategy under study for disease treatment [32, 33]. Other pathways related to Th2 cells include those involving type 2 innate lymphoid cells (ILC2s), which produce type 2 cytokines such as IL‐5 and IL‐13, promoting allergic inflammation in the respiratory mucosa [34, 35]. Experimental models have shown that these cells may contribute to chronic inflammation in allergic diseases, including rhinitis, dermatitis, and asthma, suggesting their therapeutic potential [3, 6, 36].

An emerging area of investigation highlights extracellular adenosine 5′‐triphosphate (ATP) as a central mediator of allergic inflammation. ATP can act in a purinergic receptor‐dependent manner, activating P2X and P2Y subtypes and thereby influencing immune cell recruitment, cytokine release, and epithelial permeability. In a purinergic receptor‐independent manner, it directly promotes epithelial activation and inflammatory signaling [37]. ATP‐mediated signaling has been shown to regulate eosinophil activation and other immune responses in both health and disease [38]. Experimental models have demonstrated that ATP can contribute to allergic inflammation in the nasal mucosa through multiple mechanisms, including the modulation of calcium channels and epithelial cell function [39]. The NLRP3 inflammasome is a protein complex that acts as a sensor for pathogen‐associated molecular patterns (PAMPs) and damage‐associated molecular patterns (DAMPs). ATP stimulation via purinergic receptors activates caspase‐1, leading to the maturation of IL‐1β and IL‐18 [40]. NF‐κB plays a central role in the transcription of proinflammatory genes and adhesion molecules and is essential for the expression of inflammasome components. Its inhibition is associated with reduced chronic inflammation [41].

Recent studies have expanded the understanding of the role of purinergic receptors in AR. Different subtypes of P2X (P2X_1_, P2X_3_, P2X_4_, and P2X_7_) and P2Y (P2Y_2_ and P2Y_6_) are expressed in airway epithelial and immune cells. These receptors have emerged as particularly promising targets because of their role in modulating the activation of the NLRP3 inflammasome and the release of cytokines, such as IL‐1β, which contributes to the persistence and severity of inflammation and contributes to mast cell degranulation, mucus secretion, epithelial remodeling, and Th2‐driven responses [37, 42, 43]. These findings suggest that purinergic signaling represents a promising field for therapeutic innovation in AR, with antagonists and modulators of specific subtypes under investigation as potential pharmacological strategies. Although the receptors are discussed in detail in a separate section, it is important to note that they are part of a broader inflammatory signaling network. The modulation of these pathways by natural products has received increasing attention. Compounds such as polyphenols, terpenes, and alkaloids have been shown to inhibit NF‐κB activity, reduce the expression of proinflammatory cytokines, and attenuate inflammasome activation [44–46]. These mechanisms translate to clinical benefits, such as improved symptoms in allergic and inflammatory diseases, highlighting the translational potential of these molecules in the development of new therapies.

These findings support the concept that therapies targeting molecular pathways, especially those involving ATP and purinergic receptors, may offer superior efficacy and lower toxicity, complementing or replacing conventional pharmacological approaches. Integrating natural products with the simultaneous modulation of multiple molecular targets in the inflammatory cascade has emerged as a promising strategy, expanding therapeutic options for AR. Thus, interactions between plant‐derived compounds and P2Rs represent a relevant field for translational research, with the potential to generate effective and clinically acceptable therapeutic innovations.

## 3. The P2R: Structure, Activation, and Role in Allergic Inflammation

**Figure 2:**
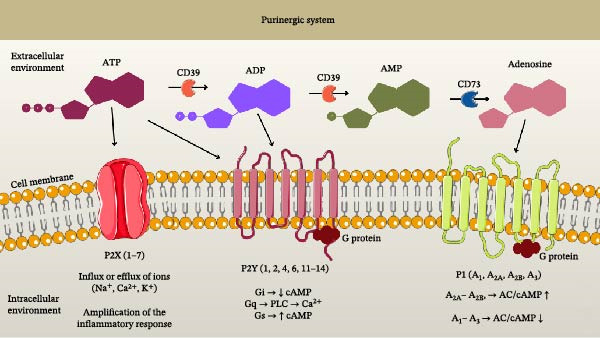
Schematic representation of the purinergic signaling system mediated by the extracellular metabolism of nucleotides and the activation of purinergic receptors. Released ATP activates P2X receptors (ion channels that mediate Na^+^, Ca^2+^, and K^+^ influx or efflux), contributing to amplification of the inflammatory response. ATP and ADP also activate P2Y receptors, which are G‐protein‐coupled receptors (GPCRs) associated with intracellular signaling pathways involving Gi, Gq, and Gs proteins, modulating cyclic AMP (cAMP), phospholipase C (PLC), and Ca^2+^ signaling. The ectonucleotidases CD39 and CD73 degrade ATP into ADP, AMP, and adenosine, respectively. Adenosine acts on P1 receptors (A_1_, A_2A_, A_2B_, and A_3_), leading to distinct immunomodulatory effects through the regulation of AC and intracellular cAMP levels (stimulated by A_2A/B_; inhibited by A_1_/A_3_), thereby modulating inflammatory signaling. The components and symbols are presented in a stylized manner. AC, adenylate cyclase; ADP, adenosine diphosphate; AMP, adenosine monophosphate; ATP, adenosine triphosphate; Ca^2+^, calcium; cAMP, adenosine monophosphate cycle; K^+^, potassium; Na^+^, sodium. Adapted from Giuliani et al. [47] and created with Servier Medical Art and Canva licensed under CC BY 3.0.

Purinergic receptors play a central role in regulating inflammatory responses and are widely distributed across immune cell types. These receptors are classified into two main subtypes: P1 receptors, which are divided into A_1_, A_2A_, A_2B_, and A_3_, with high affinity for adenosine and P2Rs, which are responsive to extracellular nucleotides (Figure 2) [28, 48]. P2Rs are subdivided into seven P2X isoforms, P2X_1_–P2X_7_, which are activated by ATP, and P2Ys, which are G‐protein‐coupled and activated by nucleotides, including ATP, adenosine 5′‐diphosphate (ADP), uracil 5′‐triphosphate (UTP), and uracil 5′‐diphosphate (UDP) [48]. Structurally, P2X receptors are trimeric ligand‐gated ion channels, with each subunit containing two transmembrane helices (TM1 and TM2), a large extracellular ATP‐binding domain, and intracellular N‐ and C‐termini. P2Y receptors, in contrast, are G‐protein‐coupled receptors (GPCRs) with seven transmembrane helices (7‐TM), reflecting their distinct signaling mechanisms [49]. Extracellular ATP acts as a DAMP, promoting inflammatory signaling, whereas adenosine exerts anti‐inflammatory effects and contributes to the resolution of the immune response. When activated by extracellular ATP released during cellular stress, tissue damage, or inflammation, P2Rs allow cation influx (Ca^2+^ and Na^+^) and K^+^ efflux, altering the membrane potential and initiating intracellular signaling cascades. In some cases, sustained P2X receptor activation can lead to the formation of large conductance pores that allow the passage of small molecules (<900 Da) and, in extreme cases, induce apoptosis or pyroptosis [50].

Several P2R subtypes are expressed in immune cells relevant to allergic inflammation, such as mast cells, eosinophils, lymphocytes, and macrophages, as well as in epithelial and endothelial cells of the airways [46]. The activation of these receptors has been associated with mast cell degranulation, cytokine secretion (including IL‐1β, IL‐6, and IL‐18), eosinophil recruitment, mucus secretion, and airway remodeling, all of which are key features of allergic diseases [48, 50]. For example, P2X_3_, P2Y_2_, and P2Y_6_ have been implicated in modulating airway immune responses and epithelial functions. In brief, studies using experimental models of allergic inflammation, such as allergen‐induced airway hyperresponsiveness or ovalbumin (OVA)‐sensitized animals, have shown that modulation of these receptors can alter inflammation and Th2‐mediated responses, supporting their functional relevance in vivo [51–53]. Furthermore, P2X_7_R, an ATP‐gated ion channel of the P2X family, is closely linked to NLRP3 inflammasome activation, triggering caspase‐1 cleavage and the maturation/secretion of proinflammatory cytokines such as IL‐1β and IL‐18, which are associated with chronic airway inflammation [54, 55]. Beyond inflammasome activation, evidence indicates that purinergic signaling is closely associated with the modulation of type 2 immune responses in allergic airway diseases. Extracellular ATP released by stressed or damaged epithelial cells acts as a DAMP capable of activating dendritic cells and promoting Th2 polarization through the induction of epithelium‐derived cytokines such as IL‐33, IL‐25, and thymic stromal lymphopoietin (TSLP) [53, 56, 57]. These mediators increase the differentiation and activation of Th2 lymphocytes, leading to increased production of IL‐4, IL‐5, and IL‐13, which are central cytokines involved in the pathophysiology of AR [57, 58]. In parallel, P2R activation contributes to eosinophil recruitment and survival by modulating cell signaling, adhesion molecule expression, and cytokine release within the airway microenvironment. ATP‐mediated activation of P2X_7_R also promotes the efflux of K^+^, a key signal for NLRP3 inflammasome assembly, resulting in caspase‐1 activation and the maturation of IL‐1β and IL‐18 [59, 60]. These cytokines further amplify Th2‐mediated inflammation and tissue remodeling, establishing a link between purinergic signaling, eosinophilic inflammation, and chronic allergic airway responses.

In experimental models of allergic inflammation, including OVA‐induced asthma and allergen‐induced rhinitis, multiple P2X and P2Y receptor subtypes are upregulated in immune and airway epithelial cells. Pharmacological inhibition or genetic deficiency of specific receptors reduces airway inflammation, hyperresponsiveness, and inflammatory mediator production, indicating their functional role in eosinophil recruitment, maintenance of Th2‐mediated responses, and chronic airway inflammation [61]. P2R activation also contributes to the resolution pathways under certain conditions. Clinical studies in asthma, a condition that often co‐occurs with AR, have demonstrated that alveolar macrophages from patients can produce proresolution lipid mediators, such as lipoxin A_4_ and resolvin D1, in response to P2X_7_ activation [62]. This suggests a dual role: amplification of certain inflammatory phases while promoting resolution under other conditions. Additionally, P2R pathways are involved in pulmonary inflammation models, such as chronic obstructive pulmonary disease (COPD) related to cigarette smoke exposure. Activation of the P2X/inflammasome pathway is correlated with increased IL‐1β levels and sustained airway inflammation, which can be attenuated with specific blockers or in receptor‐deficient mice [63].

**Figure 3:**
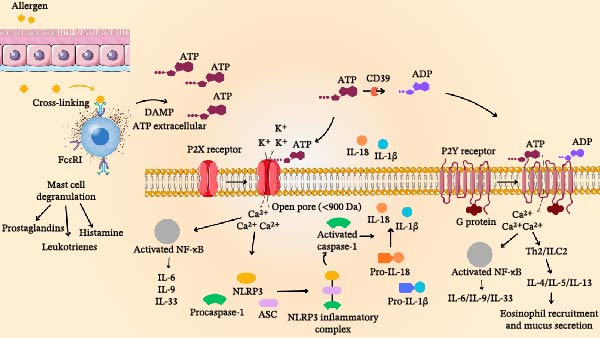
Upon allergen exposure, epithelial and immune cells release extracellular ATP, which activates P2X (ionotropic) and P2Y (metabotropic) receptors. P2X activation promotes Ca^2+^ influx and K^+^ efflux, thereby activating NLRP3 inflammasome assembly, caspase‐1 activation, and IL‐1β/IL‐18 maturation, as well as NF‐κB activation and the release of IL‐6, IL‐9, and IL‐33. In contrast, P2Y receptors modulate calcium signaling, epithelial permeability, and the release of Th2 cytokines, including IL‐4, IL‐5, and IL‐13. These cascades contribute to eosinophil recruitment, mucus secretion, and the development of chronic inflammation. Adapted from Jiang et al. [64], created with Servier Medical Art and Canva licensed under CC BY 3.0.

Taken together, these findings establish P2Rs as key modulators of allergic respiratory inflammation, representing emerging therapeutic targets in allergic respiratory diseases. Targeting multiple P2R subtypes may provide a promising strategy to modulate chronic inflammation, regulate eosinophil and mast cell activity, and balance proinflammatory and proresolution responses in AR and related airway diseases (Figure 3).

## 4. Experimental Models for the Study of AR and the P2R

Experimental models are crucial for understanding the immunological and molecular mechanisms of AR and for investigating potential therapeutic targets, such as P2X and P2Y receptors. OVA‐sensitized murine models are widely used to investigate AR pathophysiology and purinergic signaling. These models typically involve BALB/c or C57BL/6 mice that are sensitized via intraperitoneal OVA/alum injection, followed by repeated intranasal allergen challenge. Major endpoints include eosinophilic infiltration, serum IgE levels, nasal rubbing and sneezing frequency, goblet cell hyperplasia, mucus secretion, airway hyperreactivity, and Th2 cytokine production (IL‐4, IL‐5, and IL‐13) [65, 66]. These models allow the investigation of inflammatory pathways, including NF‐κB, NLRP3, and purinergic receptors, making them essential for translational studies. Importantly, the interpretation of findings involving P2Rs in AR should consider the different levels of experimental evidence currently available. In vitro studies provide important mechanistic insights into receptor activation, intracellular signaling pathways, cytokine release, and inflammatory cell responses. In vivo animal models contribute to understanding the physiological relevance of purinergic signaling in airway inflammation, eosinophilic infiltration, mucus production, and tissue remodeling. However, although these experimental approaches provide strong preclinical evidence, translational and clinical studies in human AR remain limited. Therefore, caution is needed when extrapolating mechanistic findings from cellular and animal models directly to clinical applicability, reinforcing the need for further human studies to validate the therapeutic potential of P2R modulation in AR [19, 67].

In the context of P2Rs, purinergic signaling emerges as a central axis of inflammatory amplification in the nasal mucosa. Experimental studies have demonstrated its contribution by linking extracellular ATP to NLRP3 inflammasome activation, caspase‐1 cleavage, IL‐1β release, and the amplification of inflammatory responses in allergic airway conditions [68, 69]. Recently, for greater clinical relevance, models employing environmentally relevant allergens, such as house dust mites (HDMs; *Dermatophagoides farinae/pteronyssinus*) or pollen extracts, have been developed. In these studies, BALB/c and C57BL/6 mice are typically sensitized through repeated intranasal HDM administration, and major endpoints include eosinophilic infiltration, serum IgE levels, Th2 cytokine production, goblet cell hyperplasia, nasal rubbing frequency, and airway hyperresponsiveness. These models exhibit variable responses to HDM sensitization in mice, reflecting the complex human AR pathophysiology. This is particularly relevant as it mirrors the heterogeneity observed in human AR, which comprises multiple endotypes characterized by distinct immunological profiles, including Th2 and non‐Th2 patterns, as well as differences in epithelial barrier integrity, innate immune activation, and cytokine profiles. Consequently, differences in murine responses to HDMs may reflect variations in genetic makeup, immunological polarization, and epithelial responsiveness, providing a useful, albeit simplified, framework for studying the complexity and interindividual variability of human diseases [1, 14, 70]. In these models, the P2Y_2_ receptor plays a crucial role in amplifying allergic inflammation. The activation of this receptor in epithelial and dendritic cells promotes eosinophil recruitment, proinflammatory cytokine release, and Th2 production, whereas pharmacological inhibition or deficiency of P2Y_2_R attenuates these responses, highlighting its potential as a therapeutic target in allergic airway inflammation (AAI) [71]. Complementary evidence suggests that allergen‐induced activation of epithelial P2Y_2_ receptors promotes ATP exocytosis, thereby amplifying type 2 immunity and enhancing epithelial‐driven inflammation in AR models [53]. These studies suggest that P2Y_2_ functions as a crucial amplifier of allergic inflammation, with effects that depend on the cell type and the tissue microenvironment. Different P2R subtypes have been studied to elucidate their roles in airway inflammation. P2X_4_ signaling has been implicated in airway remodeling and inflammation, with blockade attenuating eosinophilic infiltration and mediator production, supporting its role as a modulator of allergic responses [41, 72]. P2Y_6_ regulates Th2 responses through IL‐12 and IFN‐γ production in alveolar macrophages and epithelial cells, thereby limiting excessive type 2 inflammation. In contrast, P2Y_6_ inhibition exacerbates fibrosis and airway remodeling [73, 74]. Additionally, platelet P2Y_1_ signaling through the RhoA pathway has been shown to modulate leukocyte recruitment in allergic mice [75], while P2Y_12_ expression in the human nasal mucosa suggests a possible role in local inflammatory amplification [76]. In a murine AR model, P2X_7_R expressed in olfactory bulb microglia was shown to be involved in olfactory dysfunction; interventions modulating P2X_7_R altered neuroinflammation and the behavioral phenotype, highlighting the translational relevance of P2X_7_R [77]. Knockout models for inflammatory receptors such as P2X_7_ and NLRP3 inflammasome components have clarified the contributions of these pathways to rhinitis pathogenesis. Specifically, studies using P2X_7_‐deficient mice have shown that the absence of this receptor leads to a significant reduction in key inflammatory events. In these knockout models, the activation of the NLRP3 inflammasome is diminished, leading to decreased maturation and secretion of IL‐1β. Consequently, the recruitment of eosinophils is reduced, Th2 cytokine production (IL‐4, IL‐5, and IL‐13) is decreased, and airway hyperresponsiveness is attenuated [61, 78]. In the human nasal mucosa, evidence from chronic rhinosinusitis with polyps (CRSwNP) revealed P2X_7_R overexpression and increased NLRP3 and IL‐1β levels in nasal epithelial cells (HNECs) after LPS + BzATP challenge; pharmacological inhibition with the selective antagonist A740003 reduced NLRP3 and IL‐1β levels, supporting a functional link between P2X_7_R, NLRP3, and IL‐1β in the nasal mucosa and suggesting that P2X_7_ blockade may attenuate inflammasome‐dependent axes in AR [79].

These studies reinforce that P2R activation and local inflammatory stress play crucial roles in perpetuating the allergic response. Despite their major contributions, experimental murine models have translational limitations, such as differences in immune system organization, airway anatomy, allergen exposure patterns, microbiota composition, and purinergic receptor expression between mice and humans, which may influence inflammatory responses, therapeutic outcomes, and drug metabolism. Furthermore, experimental sensitization protocols using high allergen doses and adjuvants may not fully reproduce the chronic and heterogeneous nature of human AR, and the variability among mouse strains, allergen sources, and exposure pathways used can also affect the reproducibility and comparability between studies [19, 67, 80]. In parallel, while these models remain indispensable for mechanistic and pharmacological investigations, caution is needed when projecting preclinical findings directly onto the pathophysiology of human AR and therapeutic responses. Moreover, these models are useful for investigating natural products with diverse bioactive compounds possessing anti‐inflammatory and immunomodulatory properties [81, 82].

## 5. Natural Products With Activity in AR and P2X_7_ Receptor Inhibition

Various natural compounds, notably flavonoids and polyphenols, have been investigated for their anti‐inflammatory properties and therapeutic potential in AR. Experimental studies suggest that these compounds can modulate inflammatory pathways, including purinergic receptor signaling, and play crucial roles in allergic inflammation. Th2 cytokines (IL‐4, IL‐5, and IL‐13) are central to AR pathophysiology, driving IgE production, eosinophil recruitment, and mucus hypersecretion, which underpin the cardinal symptoms of nasal congestion, sneezing, and rhinorrhea. Evidence from clinical and preclinical studies highlights the importance of Th2 cells, which are successfully targeted by natural compounds [83]. A recent systematic review cataloged multiple phytochemicals effective in AR, including alkaloids and nitrogen‐containing compounds, flavonoids, phenolic acids, terpenoids, and others. These compounds, including intranasal resveratrol preparations, modulate NF‐κB, STAT6, and TLR4 and reduce IgE and Th2 cytokine levels, improving clinical outcomes in OVA models. Although most studies did not explicitly evaluate P2R, the authors highlighted the multitarget potential of these molecules, which converge on pathways influenced by P2X and P2Y signaling, including the inflammasome [84]. These effects suggest that, in addition to direct anti‐Th2 activity in RA, natural products may influence P2R‐mediated signaling given the known interplay between ATP‐activated P2Rs and inflammasome pathways. In parallel, a meta‐analysis of *Nigella* spp. Patients with AR reported improved nasal symptoms and a favorable safety profile [85]. However, although P2Rs have not been evaluated, these results support the rationale for natural drug strategies and suggest that future studies incorporate biomarkers such as P2Rs and the NLRP3 inflammasome to elucidate the underlying mechanisms involved. Mechanistic links between natural products and receptor inhibition have been established through studies directly testing the P2X_7_R. A screening of 42 polyphenols revealed that baicalein inhibited ATP/P2X_7_‐induced Ca^2+^ influx and reduced IL‐1β levels in J774.1 macrophages, confirming functional P2X_7_R antagonism. Resveratrol also has inhibitory effects, reducing the levels of inflammatory mediators such as NO, IFN‐β, and IL‐1β in LPS/ATP‐induced models. These data position polyphenols as direct modulators of P2X_7_R [86]. Recent reviews have mapped various classes of natural metabolites with P2X_7_R modulatory activity, including flavonoids, alkaloids, terpenoids, and podophyllotoxin derivatives [70], which act via pore formation blockade, ionic conductance modulation, allosteric regulation, or receptor expression reduction [87]. A relevant example is *Rheedia longifolia* (Clusiaceae) extracts and fractions, which inhibit dye uptake and P2X_7_R‐mediated ionic currents, with bisflavonoids likely being responsible [88]. Although not directly studied in AR models, these findings can be extrapolated to inflammatory conditions of the nasal mucosa. Importantly, the Chinese traditional medicine Mahuang Fuzi Xixin decoction (MFXD) has been shown to alleviate AR symptoms by inhibiting NLRP3/caspase‐1/GSDMD‐N‐mediated pyroptosis and reducing the expression of IL‐1β and IL‐18 in nasal tissue [89]. This directly connects natural product‐mediated inflammasome inhibition to a reduction in nasal mucosa inflammation, which is potentially mediated by P2X_7_ signaling, thereby linking extracellular ATP signaling, inflammasome activation, and Th2‐driven allergic responses. Other natural compounds tested in experimental AR models further support this connection [90]. Tanshinone IIA alleviates OVA‐induced AR symptoms by inhibiting Th2 cytokine production and mast cell histamine release in mice [91]. Mangiferin, a xanthone from *Mangifera indica*, reduces Th2 cytokines, eosinophil infiltration, and nasal symptoms in OVA‐induced AR models via mechanisms compatible with P2R‐mediated regulation of immune cells [92]. Flavonoids, terpenoids, and alkaloids such as warifteine and emodin reduce Th2 cytokine levels, IgE levels, eosinophil recruitment, and mast cell degranulation [91, 93, 94]. These effects are mechanistically consistent with the modulation of P2R pathways as P2X and P2Y subtypes are known to regulate mast cell degranulation, eosinophil chemotaxis, Th2 responses, and inflammasome activation. However, direct evidence linking natural product‐mediated P2R modulation to therapeutic effects in human AR remains limited. Most available studies are based on in vitro assays or in vivo *experiments* using experimental animal models, and only a few investigations have directly evaluated P2R biomarkers or signaling pathways in clinical AR settings [88–94]. Therefore, although the current findings strongly support the mechanistic plausibility of P2Rs targeting natural compounds, further translational and clinical studies are necessary to confirm their relevance in human AR.

It is important to emphasize that the therapeutic targeting of P2Rs by natural compounds must consider the context‐dependent roles of these receptors in different cell types and tissues. P2X and P2Y receptor subtypes exhibit heterogeneous expression patterns in epithelial cells, mast cells, eosinophils, dendritic cells, and macrophages, where they can perform proinflammatory or regulatory functions depending on the local microenvironment and disease stage. For example, activation of epithelial P2Y_2_ promotes ATP release and amplifies inflammation, whereas P2Y_6_ signaling in macrophages can exert protective effects by limiting excessive Th2 responses. Similarly, activation of the P2X_7_ receptor is closely linked to inflammasome activation and IL‐1β release, and these context‐dependent effects have important implications for therapeutic development. The efficacy and safety of natural compounds targeting P2Rs may depend on their ability to selectively modulate receptor activity in specific cell populations, avoiding systemic or nonspecific effects. In this sense, localized administration strategies, particularly intranasal, may offer significant advantages, allowing higher drug concentrations at the site of inflammation and minimizing systemic exposure. Furthermore, the variability in receptor expression and signaling among different inflammatory endotypes of AR suggests that personalized or endotype‐specific therapeutic approaches may be necessary to optimize clinical outcomes [53, 74, 79].

Collectively, these findings indicate that natural products can exert multitarget anti‐inflammatory effects on AR through the modulation of key pathways involved in allergic inflammation, including upstream events, such as Th2 cytokine production and mast cell activation, as well as downstream pathways involving NF‐κB signaling and NLRP3 inflammasome activation and IL‐1β production. The main natural compounds, their putative P2R targets, downstream signaling pathways, and anti‐inflammatory effects are summarized in Table 1. Although direct evidence linking many of these natural compounds to P2R modulation in AR remains limited, studies in AAI and other inflammatory disease models suggest the hypothesis that purinergic signaling may represent an upstream regulatory mechanism connecting extracellular ATP sensing to downstream inflammatory responses. Thus, natural products capable of modulating both upstream and downstream events may represent promising leads for the development of novel P2R‐targeting therapeutic profiles that are potentially advantageous for AR management.

**Table 1:** Natural compounds reported to modulate purinergic‐related and inflammatory pathways involved in allergic rhinitis and allergic airway inflammation.

Natural compound	P2 receptor subtype	Downstream targets	Observed effects	References
Baicalein	P2X_7_	Ca^2+^ influx, IL‐1β	Inhibition of P2X_7_ activation and reduced cytokine release	[86]
Resveratrol	P2X_7_	NF‐κB, IL‐1β, NO, IFN‐β	Reduction of inflammatory mediators and inhibition of P2X_7_ signaling	[84, 86]
*Rheedia longifolia* (bisflavonoids)	P2X_7_	Ionic currents, pore formation	Inhibition of dye uptake and receptor‐mediated currents	[88]
Mangiferin	Indirect (P2‐related pathways)	Th2 cytokines (IL‐4, IL‐5, IL‐13)	Reduced eosinophilia and allergic inflammation	[92]
Tanshinone IIA	Indirect (P2‐related pathways)	Histamine release, Th2 cytokines	Suppression of mast cell activation and cytokine production	[91]
Warifteine	Indirect (P2‐related pathways)	IgE, eosinophil recruitment	Reduction of allergic response markers	[93]
Emodin	Indirect (P2‐related pathways)	Mast cell degranulation, cytokines	Anti‐inflammatory and antiallergic effects	[94]
Mahuang Fuzi Xixin decoction	P2X_7_/NLRP3 axis (indirect)	NLRP3, caspase‐1, IL‐1β, IL‐18	Inhibition of inflammasome activation and pyroptosis	[89]

## 6. Challenges and Future Perspectives for Natural Product‐Based Therapies and P2X_7_ Modulation

Combining natural compounds with anti‐inflammatory properties and the ability to modulate P2R offers a promising approach for AR treatment. However, multiple P2R subtypes, including P2X_3_, P2X_4_, P2X_7_, P2Y_2_, and P2Y_6_, have been detected in immune and airway epithelial cells, revealing their role in modulating inflammatory and allergic cascades in AR models and demonstrating the potential of natural products that act on purinergic receptors and related allergic and inflammatory pathways. However, evidence for P2R modulation by natural products in AR remains limited. Although numerous natural compounds, such as flavonoids, polyphenols, terpenoids, and alkaloids, have demonstrated anti‐inflammatory and antiallergic effects on AR, few studies have specifically explored whether these effects are mediated through P2Rs. This represents a significant gap as the multitarget potential of these compounds could involve modulation of both P2X and P2Y receptors, contributing to the observed reduction in Th2 responses, eosinophil infiltration, mast cell activation, and inflammasome‐mediated cytokine release [48, 50, 61]. Most studies involving receptors and natural compounds have been conducted in general inflammation or other respiratory disease models, necessitating specific investigations with evidence directly linked to human or animal AR models to validate their clinical relevance [46, 95, 96].

Moreover, standardization of plant extracts and bioactive fractions is crucial as variations in chemical composition may significantly affect activity. Pharmaceutical strategies, such as microencapsulation, are also essential to ensure reproducibility and therapeutic relevance, and innovative formulations should be evaluated in models that faithfully reflect the AR pathophysiology.

From a translational perspective, AR‐targeted trials using natural compounds and directly evaluating P2Rs are nearly nonexistent, representing a critical knowledge gap. Future studies should prioritize not only the clinical efficacy but also the molecular mechanisms, pharmacokinetics, and pharmacodynamics of P2R‐modulating natural products. Filling these gaps will provide a clearer understanding of the translational potential of natural products as P2R‐targeting therapies and may lead to the development of innovative therapies offering safe and effective alternatives for AR patients who do not respond adequately to conventional approaches. Consolidating P2Rs as emerging targets in AR via the use of natural products as potential pharmacological agents.

### 6.1. Clinical Advances and Therapeutic Perspectives

Purinergic signaling has increasingly been recognized as a promising therapeutic target. Despite advances in the identification of select antagonists, their clinical translation remains limited, particularly in the context of allergic airway diseases. Evidence from preclinical studies has shown that purinergic modulators can be successfully delivered through intranasal and inhalation formulations and that they are used mainly as P2Y_2_ agonists. For example, the P2Y_2_ agonist denufosol tetrasodium (INS37217) was tested in cystic fibrosis patients as an inhaled spray in clinical studies of safety, tolerability, and airway effects, serving as a translational proof‐of‐concept for purinergic drug delivery through the respiratory route [97–99]. Although no published studies have described P2X_7_ antagonists formulated as nasal sprays or aerosols in animals or humans, preclinical protocols using intranasal or intratracheal instillation of ATP have demonstrated that the respiratory route can activate and modulate P2X_7_ in vivo, validating the technical plausibility of intranasal formulations targeting this receptor [100]. Intranasal delivery presents pharmacotechnical advantages, including rapid onset, avoidance of first‐pass metabolism, and the potential for localized modulation of mucosal inflammation [101, 102]. The broader literature concerning intranasal formulations provides consolidated technological guidance, suggesting that developing nasal sprays for P2X_7_ antagonists is feasible but requires rigorous evaluation of physicochemical compatibility, local toxicity, stability, and dose efficacy. Patent filings describing intranasal purinergic modulators such as WO2022087174A1 for suramin highlight the growing industrial and translational interest in this therapeutic avenue [103]. Furthermore, systemic trials of P2X_7_ antagonists, such as the compound AZD9056, which was evaluated in a randomized phase II trial in rheumatoid arthritis, showed good safety and tolerability but limited efficacy, with no significant improvement in the ACR20 response (American College of Rheumatology 20% response) or inflammatory biomarkers compared with placebo [104].

It is important to emphasize that a critical gap still exists between systemic P2X_7_ antagonism and its potential application in localized airway administration. Clinical trials with P2X_7_ antagonists in systemic inflammatory diseases, such as rheumatoid arthritis, have demonstrated favorable safety profiles but limited therapeutic efficacy, suggesting that systemic blockade may be insufficient to modulate the specific inflammatory microenvironments of each tissue. In contrast, AR is characterized by mucosal inflammation, in which extracellular ATP gradients, epithelial barrier dysfunction, and compartmentalized immune responses play central roles. Therefore, the pharmacokinetic and pharmacodynamic requirements for effective P2X_7_ modulation in the nasal mucosa may differ substantially from those observed under systemic conditions. Intranasal administration could, in principle, achieve higher local drug concentrations while minimizing systemic exposure. However, this approach introduces additional challenges, including mucociliary clearance, limited residence time, epithelial permeability, and formulation stability [105–107]. Furthermore, achieving ideal pharmacokinetic profiles in the nasal mucosa remains a challenge as rapid elimination and enzymatic degradation can reduce drug bioavailability at the target site [105]. Furthermore, the high structural similarity between P2R subtypes and their wide tissue distribution complicates the development of highly selective antagonists, increasing the risk of off‐target effects and limiting therapeutic precision [108–111]. In addition, the heterogeneous expression of these receptors in epithelial and immune cell populations in the nasal mucosa may influence the therapeutic response and contribute to variable clinical outcomes as P2X and P2Y signaling are differentially regulated across mucosal cell types and inflammatory endotypes [24, 50, 53]. These considerations highlight that the successful use of P2R antagonists for the treatment of AR will require not only reformulation strategies but also a deeper understanding of the dynamics of local purinergic signaling and optimized delivery systems adapted to the airway microenvironment.

In summary, further preclinical and clinical investigations are needed to validate the therapeutic potential of P2R antagonists in airway inflammation. Emphasis should be placed on the development of locally active pharmaceutical formulations, such as intranasal formulations, which may provide more effective and targeted modulation of purinergic signaling in AR.

## 7. Conclusion

P2Rs, including both the P2X and P2Y subtypes, play central roles in AR by regulating mast cell degranulation, Th2 cytokine release, eosinophil recruitment, and NLRP3 inflammasome‐mediated inflammation. Natural products, such as polyphenols, flavonoids, terpenoids, and alkaloids, exhibit multitarget anti‐inflammatory and antiallergic effects in AR, many of which are consistent with the modulation of P2R pathways, which reduce inflammation and protect the nasal mucosa.

However, despite this potential, direct evidence for P2R‐targeted effects of natural products on AR is still scarce, highlighting a critical gap in research. Addressing this through carefully designed preclinical and clinical studies will not only clarify the mechanisms of action but also validate the development of new therapeutic strategies for AR.

## Funding

This study was supported by the National Council for Scientific and Technological Development (CNPq), Brazil (Grant 130703/2025‐7) (Robson Xavier Faria holds a grant with Fellowship Process Number 302890/2025‐4) and the FAPERJ (Research Support Foundation of the State of Rio de Janeiro) with Grant E‐26/200.533/2026.

## Conflicts of Interest

The authors declare no conflicts of interest.

## Data Availability

No new data were generated in this study. All information supporting the conclusions of this article is available in the cited references.
